# Virulent *Brucella nosferati* infecting *Desmodus rotundus* has emerging potential due to the broad foraging range of its bat host for humans and wild and domestic animals

**DOI:** 10.1128/msphere.00061-23

**Published:** 2023-07-05

**Authors:** Gabriela Hernández-Mora, Carlos Chacón-Díaz, Andres Moreira-Soto, Osvaldo Barrantes-Granados, Marcela Suárez-Esquivel, Eunice Viquez-Ruiz, Elías Barquero-Calvo, Nazareth Ruiz-Villalobos, Daniela Hidalgo-Montealegre, Rocío González-Barrientos, Elena A. Demeter, Josimar Estrella-Morales, Ana-Mariel Zúñiga-Pereira, Carlos Quesada-Gómez, Esteban Chaves-Olarte, Bruno Lomonte, Caterina Guzmán-Verri, Jan Felix Drexler, Edgardo Moreno

**Affiliations:** 1 Unidad de Microbiología Médico Veterinaria, Servicio Nacional de Salud Animal (SENASA), Ministerio de Agricultura y Ganadería, Heredia, Costa Rica; 2 Centro de Investigación en Enfermedades Tropicales (CIET), Facultad de Microbiología, Universidad de Costa Rica, San José, Costa Rica; 3 Charité–Universitätsmedizin Berlin, Corporate Member of Freie Universität Berlin, Humboldt-Universität zu Berlin, and Berlin Institute of Health, Institute of Virology, Berlin, Germany; 4 Programa de Investigación en Enfermedades Tropicales (PIET), Escuela de Medicina Veterinaria, Universidad Nacional, Heredia, Costa Rica; 5 Section of Anatomic Pathology, Department of Biomedical Sciences, College of Veterinary Medicine, Cornell University, Ithaca, New York, USA; 6 Instituto Clodomiro Picado, Facultad de Microbiología, Universidad de Costa Rica, San José, Costa Rica; University of Kentucky College of Medicine, Lexington, Kentucky, USA

**Keywords:** *Desmodus*, vampire bats, prey preference, bats, *Brucella*, brucellosis, pathogen, zoonosis, bacteria

## Abstract

*Desmodus rotundus,* vampire bats, transmit dangerous infections, and brucellosis is a hazardous zoonotic disease, two adversities that coexist in the subtropical and tropical areas of the American continent. Here, we report a 47.89% *Brucella* infection prevalence in a colony of vampire bats inhabiting the tropical rainforest of Costa Rica. The bacterium induced placentitis and fetal death in bats. Wide-range phenotypic and genotypic characterization placed the *Brucella* organisms as a new pathogenic species named *Brucella nosferati* sp. nov., isolated from bat tissues, including the salivary glands, suggesting feeding behavior might favor transmission to their prey. Overall analyses placed *B. nosferati* as the etiological agent of a reported canine brucellosis case, demonstrating its potential for infecting other hosts. To assess the putative prey hosts, we analyzed the intestinal contents of 14 infected and 23 non-infected bats by proteomics. A total of 54,508 peptides sorted into 7,203 unique peptides corresponding to 1,521 proteins were identified. Twenty-three wildlife and domestic taxa, including humans, were foraged by *B. nosferati*-infected *D. rotundus*, suggesting contact of this bacterium with a broad range of hosts. Our approach is appropriate for detecting, in a single study, the prey preferences of vampire bats in a diverse area, demonstrating its suitability for control strategies where vampire bats thrive.

**IMPORTANCE:**

The discovery that a high proportion of vampire bats in a tropical area is infected with pathogenic *Brucella nosferati* and that bats forage on humans and many wild and domestic animals is relevant from the perspective of emerging disease prevention. Indeed, bats harboring *B. nosferati* in their salivary glands may transmit this pathogenic bacterium to other hosts. This potential is not trivial since, besides the demonstrated pathogenicity, this bacterium possesses all the required virulent arsenal of dangerous *Brucella* organisms, including those that are zoonotic for humans. Our work has settled the basis for future surveillance actions in brucellosis control programs where these infected bats thrive. Moreover, our strategy to identify the foraging range of bats may be adapted for exploring the feeding habits of diverse animals, including arthropod vectors of infectious diseases, and therefore of interest to a broader audience besides experts on *Brucella* and bats.

## INTRODUCTION

Costa Rica comprises ~6% of the world’s biodiversity, bats being the country’s second most varied mammal group, with 112 out of 1,400 known species worldwide. Although most bats are beneficial and ecologically relevant, the blood-feeding *Desmodus rotundus* vampire bat is considered a plague because it is a reservoir and vector of a collection of reemerging and emerging microbial pathogens (1 - 6). *D. rotundus* is found below 1,500 m of altitude in the tropical and subtropical areas of the Americas, with an increasing range toward the Northern hemisphere (7, 8) attributed to climate change (9). Vampire bat populations have drastically increased due to livestock farming, their primary food source (6, 10 - 12). Consequently, most Latin American countries control them through extermination strategies (13, 14).

*D. rotundus* bats bite their prey directly above the blood vessels, have anticoagulants in their saliva to aid blood flow, and use their tongue to sip blood (12, 15, 16). One prey per bat is generally observed; however, several vampire bats can forage on a single animal (12) and even feed on the same prey on various occasions (17). Through this process, they transmit dangerous infectious diseases (1 - 6). After feeding, the vampire bat flies back to its colony in man-made or natural structures, with colonies ranging from ten to hundreds (16, 18). These chiropterans are highly social animals that commonly regurgitate blood meals to feed each other (19). Through many decades of observation, it has been demonstrated that vampire bats forage from diverse prey, including domestic and wildlife animal species (6, 11, 12, 15, 16). Humans are also foraged by *D. rotundus*; however, the frequency and the number of cases may be underreported because most incidents occur in rural landscapes (10).

Brucellosis is a zoonotic bacterial infection of veterinary and public health relevance (20). In Costa Rica, nearly 11% of all bovine herds have brucellosis, causing a high economic loss in farm animals and a zoonotic risk (21, 22). Antibodies against *Brucella* organisms have been found in 9.4% of vampire bats in Brazil (23). In addition, *Brucella suis* biovar 5, closely related to those isolated from rodents, has been identified in insectivorous bats from the Caucasus (24, 25). Likewise, a new species of *Brucella* organism has been isolated from humans working in the Amazonian rainforest in French Guiana, from which the infection source remains unknown (26).

Here, we describe the prey preferences of *D. rotundus* vampire bats infected with a new species of virulent *Brucella* organism in a region of the tropical rain premontane forest of Costa Rica and rural surroundings. This new species, named *B. nosferati* sp. nov., is a primary pathogen of bats that resides in different organs, including the salivary glands, with the potential to infect other mammals. The colony of *D. rotundus*-infected bats studied foraged on a wide range of domestic and wildlife animals and humans, which may become hosts of this newly discovered pathogenic bacterial species causing brucellosis.

## RESULTS

### Study site and bat colony characteristics

**Fig 1:**
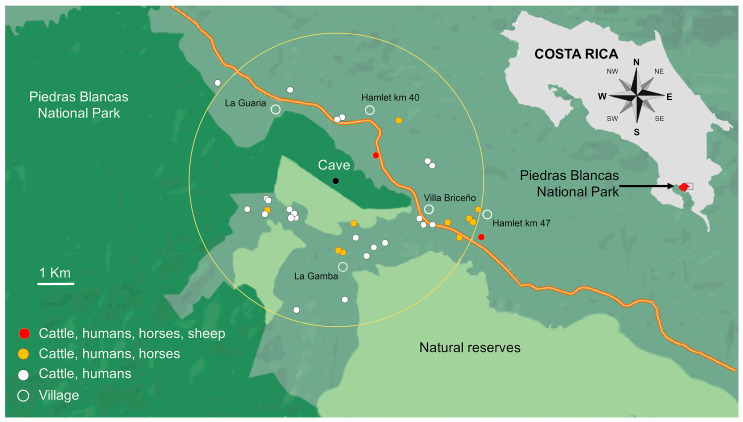
Map of the eastern region of Piedras Blancas National Park, natural reserves, and the rural and suburban surroundings. In the upper right of the scheme is the map of Costa Rica, indicating the localization of the Park in red; the black square insert depicts the area of study amplified to the left of the map. The park (in deep emerald green to the left of the map) and the boundary of natural reserves (in lime green) in the furthermost East region of the park are surrounded by extensive plantations of oil palm trees, farms, a few small villages, and secondary forest (in pistachio color, mainly in the center and right area of the map). The rest of the park (138 km^2^) extends to the West (partially shown). The cave’s location with the colony of *D. rotundus* studied is within the park and indicated by a full black circle in the middle of a larger circle depicting a radius of 4 km. The open circles indicate the villages, while the white, yellow, and red circles show the locations of livestock farms within vampire bats foraging range of ~4 km of the cave. Wildlife mainly inhabits the park and the natural reserves, with sporadic intrusion in semi-rural areas. Domestic animals seldom enter the park or natural reserves. A thick yellow line indicates the Inter-American Highway. The coordinates of the cave are 8°43*'*40.1*''*N and 83°11*'*15.2*''*W.

Seventy-one vampire bats (43 female and 28 male) were collected in a cave adjacent to the National Park Piedras Blancas of Costa Rica (141.2 km^2^), located in the far eastern region of the park. All vampire bats were negative for the presence of rabies and coronavirus. Fifty-nine were adults, six were subadults, four were juveniles, and two were newborn bats. In addition, seven pregnant bat females and the corresponding fetuses were included in the study (Dataset S1). The cave of the *D. rotundus* colony is in a tropical premontane rainforest area (Fig. 1). According to the census and the annual survey of the Animal Health Service (SENASA) of Costa Rica (Dataset S1), all surrounding farms within a radius of ~4 km possess cattle; some have horses, two farms have sheep, and three have poultry for commercial purposes. Most farms have a few chickens and ducks (~5), dogs (~2), and cats (~1) roaming around the shelter. None of the farms in a range of 8 km possess pigs. In the park territory, there are nearly 100 species of wild mammals and over 400 bird species (27 - 32).

### Identification of pathogenic *Brucella* organisms in bats

Since vampire bats feed on cattle, and bovine brucellosis is highly prevalent in Costa Rica (21), we anticipated these chiropterans could be infected with *Brucella* since this is a blood-borne pathogen circulating within cells (20). Using serological assays, we found that 33.8% (24/71) of the bats had antibodies against the *Brucella* lipopolysaccharide (LPS) (Table 1), a higher proportion than the 9.4% reported in Brazil (23). So far, the only *Brucella* species identified in Costa Rica’s livestock is *Brucella abortus* (21, 22). Since bovines are the most common prey of vampire bats, we hypothesized that bats could become infected with this bacterium.

**TABLE 1:** Isolation of *B. nosferati* and detection of antibodies against *Brucella* LPS in *D. rotundus*

Bacterial culture	Serology^*a*^	Positive	Negative	Prevalence %
Positive	Positive	6	−	8.46
	Negative	4	−	5.60
	Not performed^*b*^	7	−	9.89
Negative	Positive	17	−	23.95
	Negative	−	15	−
	Not performed^*b*^	−	22	−
Accumulated prevalence %		47.9	−	47.9

^*a*^ RBT/cELISA.

^*b*^ Not enough serum to perform tests.

**Fig 2:**
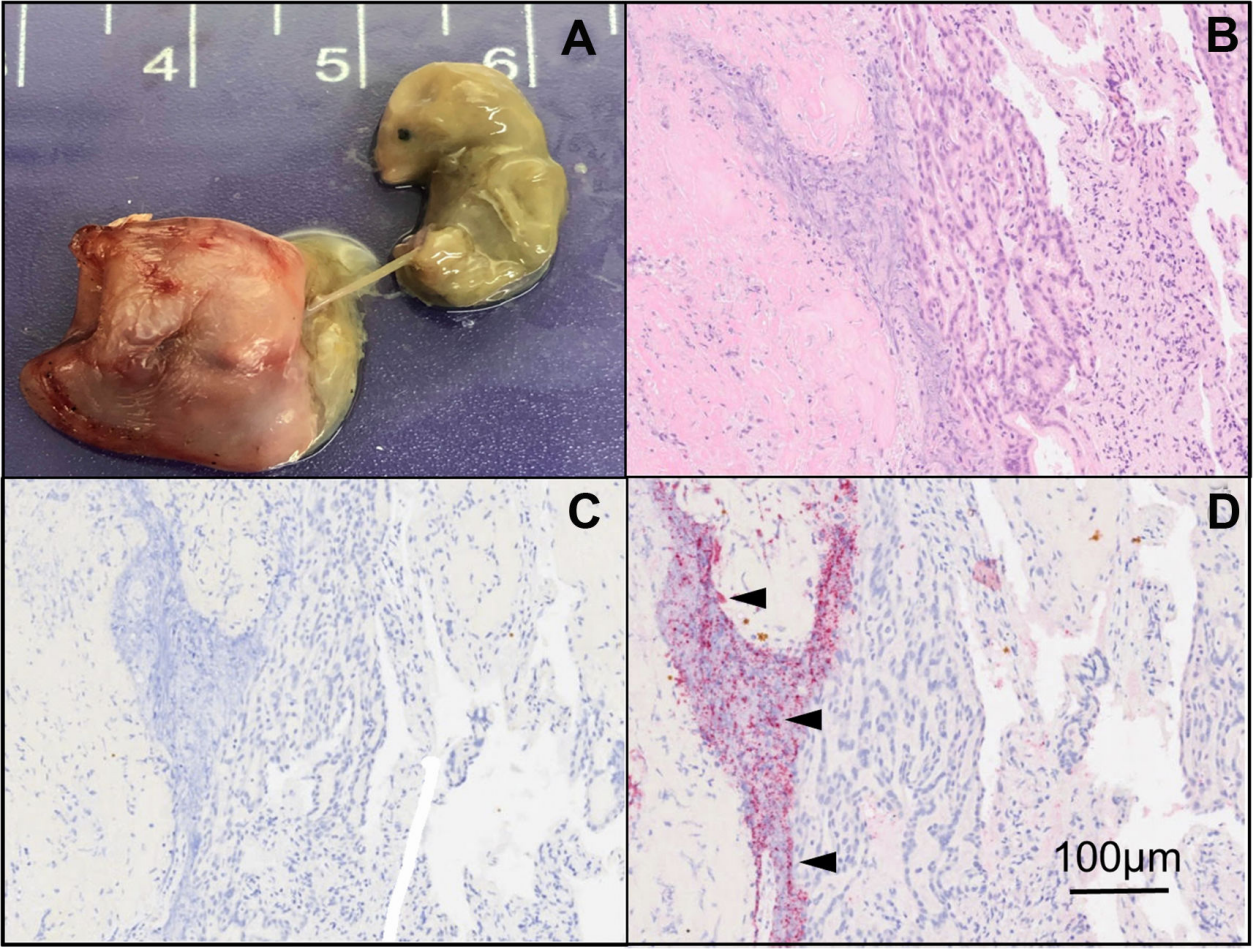
*D. rotundus* placenta infected with *B. nosferati*. Uterus with serosal congestion and mild focal hemorrhage, necrosuppurative endometrial exudate, placentitis, and dead fetus of *D. rotundus* infected with *B. nosferati* (**A**). Hematoxylin and eosin stain of placental serial sections with hemodichorial and labyrinth zone necrosis of double-layered trophoblasts and placental stroma with replacement by karyorrhectic cellular debris, conglomerates of fibrin, and degenerate leukocytes (left side of image), adjacent to better preserved double-layered trophoblasts (center) and conglomerates of variably degenerate leukocytes (**B**). Negative immunoperoxidase control (**C**). Immunoperoxidase detection of *Brucella* organisms (arrowheads), with strong positive immunolabeling in a focal area of necrotic double-layered trophoblasts, karyorrhectic cellular debris, and degenerate leukocytes (**D**).

Most of the vampire bats did not reveal noticeable internal or external lesions. However, the gross anatomical and histological examination and immunohistochemistry of 4 pregnant vampire bats out of 16 showed a severe necrosuppurative placentitis with a mummified fetus characterized by necrosis of double-layered trophoblasts, numerous degenerate leukocytes, and karyorrhectic cellular debris characteristic of brucellosis (33). Strong *Brucella* sp. immunolabeling was associated with necrosis and inflammation of the double-layered trophoblasts, demonstrating extensive intracellular bacterial parasitism (Fig. 2). Following this, we attempted isolation and characterization of these pathogenic bacteria.

*Brucella* organisms were isolated in 6 male and 11 female bats, of which one was a nursing newborn male and one juvenile male (Dataset S1). As in ruminants, the bacterium was isolated from the infected bats’ placenta, fetus, milk, and other tissues (Table 2). *Brucella* organisms were also isolated from the salivary glands of 11.8% of the infected bats, an unprecedented finding in brucellosis. This event suggests that transmission through the bats’ feeding habits might be plausible to other mammals since the minimal infectious dose for pathogenic *Brucella* organisms has been estimated from 5 to 50 bacteria (34).

**TABLE 2:** *B. nosferati* in organs of *D. rotundus* and the performed genetic analysis of the isolates

Isolated from	Culture positive	*Brucella*-ladder	MLVA-16^*a*^	WGSA
Mammary gland	1	1	1	1
Uterus	1	1	1	1
Kidney	2	2	1	0
Placenta	2	2	2	2
Salivary gland	2	2	2	2
Fetus	3	3	2	2
Lung	4	4	2	1
Brain	6	6	5	6
Liver	6	6	3	0
Swab of tissues	7	7	4	0
Intestine content	8	8	4	5
Total	42	42	27	20

^*a*^ Multiple Locus Variable-number Tandem Repeat Analysis-16 (MLVA).

**Fig 3:**
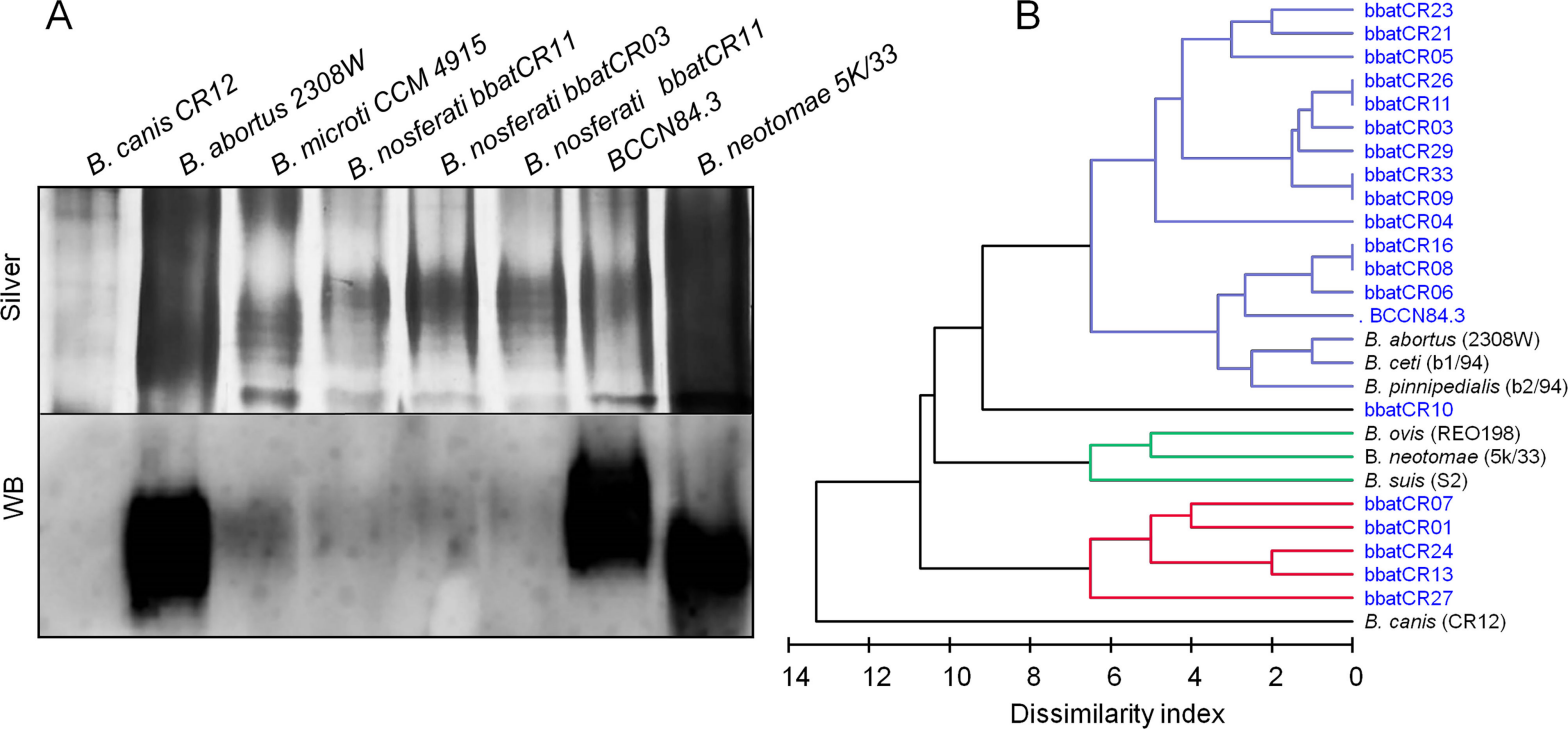
Characterization of major outer membrane lipids of *B. nosferati*. Silver-stained SDS-PAGE and Western blotting of *Brucella* LPS preparation from different species and strains (**A**). Dendrogram based on the similarity of FAMEs profiles of different *Brucella* species (**B**).

The bacteriological characterization showed that the bat isolates were smooth brucellae resembling the pathogenic *Brucella* sp. BCCN84.3 isolated from a dog orchiepididymitis case in Costa Rica (35, 36), except that the *Brucella* of the bat strains were dominant M type while the BCCN84.3 strain was dominant A type (Table 3). The dominant M profile of the bat isolates was confirmed through SDS-PAGE/Western blotting of the LPSs using *B. microti* (dominant M) and *B. abortus* (dominant A) LPSs as controls. The subtle laddering pattern of LPSs of the *B. nosferati* strains revealed predominantly M-type LPS. Likewise, the strong antibody reaction with A>M specificity against the LPS of BCCN84.3 showed that this bacterium had predominantly A-type LPS, in contrast to the milder reaction displayed by the M-type LPSs of the bat isolates (Fig. 3A).

**TABLE 3:** Microbiological characterization of *B. nosferati* and comparison with *Brucella* reference strains

Strains^*a*^	CO_2_ requirements	Nitrate reduction	Oxidase	Urease^*b*^	Orange acridine agglutination	Serum agglutination against^*c*^		Growth on dyes mg/mL^*d*^			
								Thionin		Basic fuchsin	
						A	M	10	20	10	20
*B. nosferati*	No	V^*e*^	+	+	−	−	+	+	+	+	+
*Brucella* sp. BCCN84.3	No	V	+	+	−	+	−	+	+	+	+
*B. abortus* 2308W	No	-	+	+	−	+	−	−	−	+	+
*B. suis* 1330	No	+	+	+	−	+	−	+	+	−	−
*B. melitensis* 16M	No	+	+	+	−	−	+	+	+	+	+
*B. ovis* 63/290	Yes	−	+	−	+	−	−	+	−	−	−
*B. canis* bcanCR12	No	+	+	+	+	−	−	+	+	−	−
*B. neotomae* 5K/33	No	+	−	+	−	+	−	−	−	−	−
*B. microti* CCM 4915	No	−	+	+	−	−	+	+	+	+	+
*B. ceti* B1/94	No	+	+	+	−	+	−	+	+	+	+
*B. pinnipedialis* B2/94	Yes	+	+	+	−	+	−	+	+	+	+

^*a*^ All strains were non-motile aerobic Gram-negative coccobacilli or short rods ~0.6 mm in diameter and ~0.6–1.5 mm in length produced H_2_S, catalase positive, indole negative, citrate negative, negative for gelatin liquefaction, and do not produce acid from carbohydrates in conventional media.

^*b*^ *B. nosferati* urease was active in ≤20 minutes, *Brucella* sp. BCCN84.3 urease was active at ≥120 minutes.

^*c*^ Serum against “A” and “M” LPS epitopes were determined by agglutination with monospecific serum.

^*d*^ Dye concentrations expressed in micrograms per milliliter of culture medium and plates incubated under 10% CO_2_ atmosphere.

^*e*^ Variable result "V".

**Fig 4:**
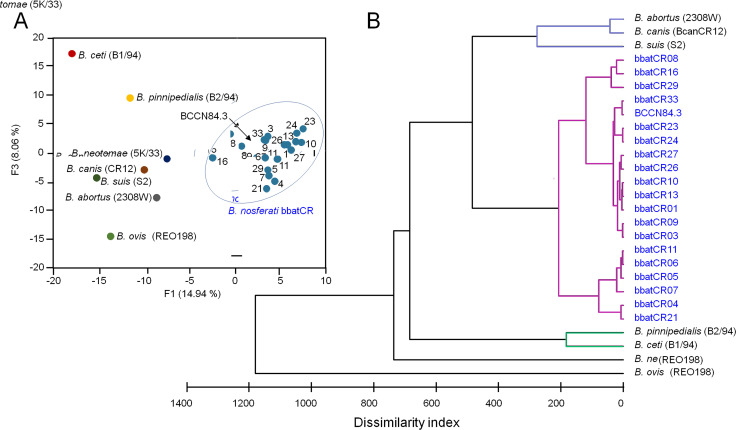
MALDI-TOF proteomic analysis of various *Brucella* species and strains. Two-component analysis reveals that the proteomes of *B. nosferati* and BCCN84.3 strains are closer among them (blue dots within a circle) and apart from other *Brucella* species (**A**). Dendrograms based on MALDI-TOF analysis revealed that all *B. nosferati* and BCCN84.3 isolates are grouped in a distinctive cluster. (**B**) For bacterial codes, see Dataset S2.

The biochemical profile of the *Brucella* vampire bat strains was characterized by the activity of Ala-Phe-Pro-arylamidase, β-N-acetyl-glucosaminase, L-proline arylamidase, tyrosine arylamidase, α-glucosidase, and urease and by the utilization of D-glucose, D-maltose, palitose, sucrose, and D-trehalose and the alkalinization of L-lactate and succinate (Dataset S2). The fatty acid methyl ester (FAMEs) profile revealed some clustering of the bat strains. However, several of them were intertwined with other *Brucella* species (Fig. 3B). All the *Brucella* vampire bat isolates had the characteristic long-chain fatty acids and lactobacillic acid of the classical virulent *Brucella* (Dataset S2). The matrix assisted laser desorption/ionization time-of-flight mass spectrometry (MALDI-TOF) proteomic analysis demonstrated that the *Brucella* bat isolates and the etiological agent of canine orchiepididymitis, the BCCN84.3 strain, were in the same cluster, separated from other brucellae (Fig. 4). Although the bacteriological profile and mass spectrometry analysis suggested a different *Brucella* species, the results were not definitive. These properties caught our attention since they did not match the *Brucella* organisms isolated in Costa Rica (22).

### Phylogenetic characterization of *Brucella* isolates

**Fig 5:**
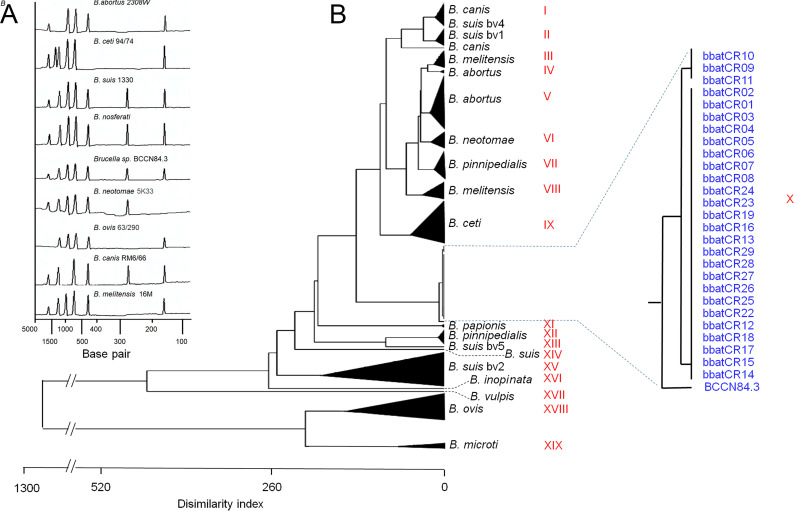
Typification of *B. nosferati* strains by Bruce-ladder and MLVA-16 profiles of Bruce-ladder scanned bands of different *Brucella* species (**A**). Dendrogram based on MLVA-16 analysis of *Brucella* species and strains (**B**). The values obtained for each MLVA-16 marker are in Dataset S3 and uploaded to microbes genotyping (https://microbesgenotyping.i2bc.paris-saclay.fr/databases/public) under the name *“Brucella* v4_6_5” dataset. For bacterial clusters (in Roman numbers) and bacterial codes, see Dataset S3.

The *Brucella* bat and BCCN84.3 strains displayed a Bruce-ladder profile indistinguishable from that of *B. suis* 1330 strains (Fig. 5A). However, the variable number of tandem repeats-16 loci (MLVA-16) analysis resolved that the bat and BCCN84.3 isolates clustered apart from other *Brucella* species (Fig. 5B). Likewise, the phylogenetic analysis of the *omp2a* and *omp2b* porin genes showed that *Brucella* bat and BCCN84.3 isolates grouped in a distinct cluster from other *Brucella* species (Fig. S1), displaying a recombination event in a region close to the 5′, which is identical to the omp2b porin sequence.

**Fig 6:**
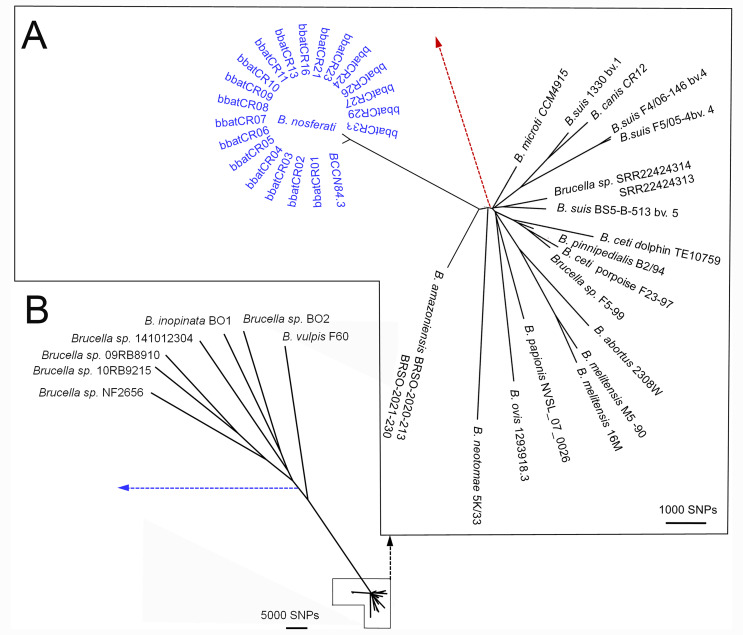
Phylogenetic relationship of *B. nosferati* and BCCN84.3 strains with other *Brucella* species. Classical *Brucella* (**A**) and nonclassical *Brucella* (**B**). Forty-nine *Brucella* and two *Ochrobactrum* species were used for the phylogenetic reconstruction. The total alignment length was 3,316,799 bp, and 558,949 SNPs were used for the reconstruction. All branching points have bootstrap values above 85. Segments of the tree were magnified through Dendroscope version 3.5.8 to increase resolution; the scale is indicated next to each magnified region. The dotted red line indicates the connection with nonclassical brucellae, while the dotted blue line is the connection with the *Ochrobactrum* genus (not shown). *B. nosferati* and BCCN84.3 are colored in blue. For bacterial strains and codes, see Dataset S3.

Among the *Brucella* genomes, 210,174 single-nucleotide polymorphisms (SNPs) were used for whole-genome sequence (WGSA) phylogenetic analyses (Dataset S3), showing that the bat strains clustered tight in a branch far from other brucellae. The closest relative was *B. amazoniensis* isolated in the rainforest of French Guiana (Fig. 6). The number of SNP differences between the *Brucella* bat and the BCCN84.3 strain ranged from 80 to 150, significantly less than those described in other *Brucella* species. For instance, the *Brucella* bat and *Brucella* sp. BCCN84.3 isolates, compared with *B. amazoniensis* and *B. suis* 1330 strain, differed in 2,378–2,387 and 7,159–7,194 polymorphic sites, respectively. From these, 6,025 are in coding region 196 with a dN/dS ratio of 0.54 (*P*-value < 0.0001) (Dataset S3). These analyses demonstrated that the bat isolates and the *Brucella* sp. BCCN84.3 strain belonged to the same distinctive classical species of *B. nosferati*, despite being isolated years apart from different hosts. Following a taxonomical approach, we named the bat isolates *Brucella nosferati* sp. nov., reminiscent of the Nosferatu vampire legends of Transylvania. From the genomic and bacteriological perspectives, the pathogenic *Brucella* sp. BCCN84.3 strain belonged to the same species and, from now on, the type strain named *B. nosferati* BCCN84.3, the first species of this group isolated in Costa Rica (35, 36).

According to the WGSA, *B. nosferati* had two chromosomes with no plasmids, nine IS711 elements, recent recombination events, and a similar number of anomalous regions shared with other classical brucellae (Fig. S2), however, with a distinct lineage and genomic islands (Fig. S3). Likewise, the WGSA showed all the genes that encode virulence factors such as VirB operon (BAW_20058 to BAW_20068), VjbR (BAW_20116), two-component system BvrR/BvrS (BAW_12006 to BAW_12007), β-cyclic glucan (BAW_11641), and flagellum-like system (BAW_11648 to BAW_11649) are conserved in *the Brucella* bat isolates. Genes coding for the toll-interleukin-1 receptor domain-containing protein BtpA predicted as a VirB effector (BAW_10265) and putative integrases (BAW_10237, BAW_10274) were absent. Likewise, the manBOAg (BAW_10538), tentatively involved in the LPS-core mannose synthesis, was 48 bp, which corresponds to the size of *B. ovis* (BOV_0540) and *B. abortus* 2308W (BAW_10538), but it was shorter than the corresponding *B. melitensis* (BMEI1396). It is clear that *B. nosferati* is a classical brucellae equipped with all the virulence arsenal similar to the most pathogenic *Brucella* species (20, 37).

### The *D. rotundus* foraging prey taxa

**Fig 7:**
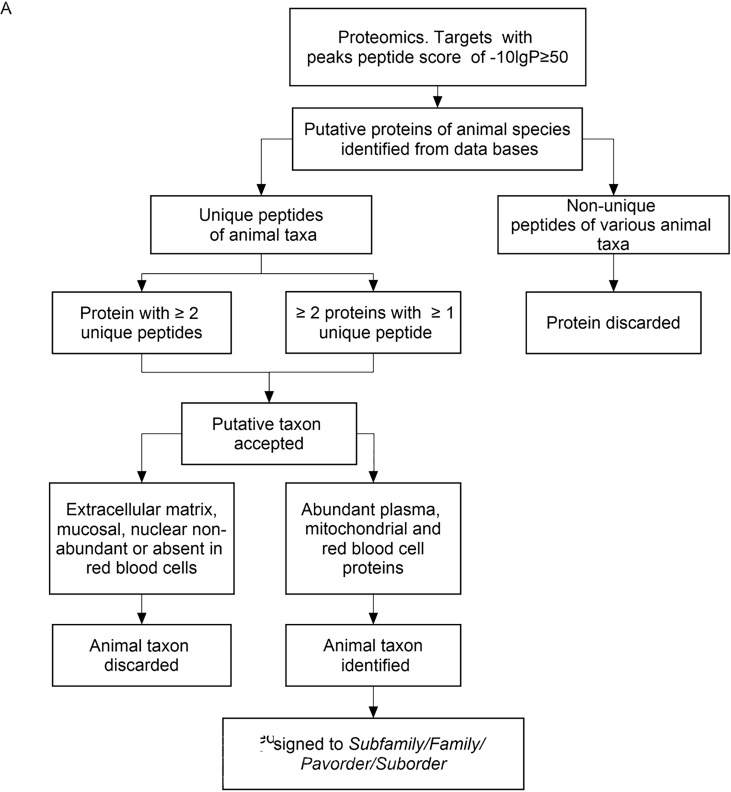
Flowchart of the strategy followed to curate the peptide data for selecting the proteins of a given taxon group. For details of the selected proteins, see Dataset S1. Except for pigeons and squirrels, which are abundant in the park and surroundings, unlikely foraging vampire prey, such as chiropterans, animals of less than 600 g, aquatic birds and mammals, raptor birds, and nocturnal birds, were excluded from the analysis. *D. rotundus* proteome was included as a control for excluding peptides.

Since *B. nosferati* was found in the salivary glands of 11.8% of the infected bats, we decided to explore the foraging prey range through proteomics of the bat’s intestinal contents. Vampire bats generally travel no more than ~4 km from their lair. However, we estimated the presence of bird and mammal species as potential prey at a radius of 8 km of the bats’ cave. This range is the average maximum distance that *D. rotundus* may fly from their lair in 1 day (18) and a significant distance for the mobility of the animal species within the examined area. The study included 2 subfamilies, 18 families, 1 parvorder, and 3 suborders representing 49 mammal and 10 bird species, which were the most likely prey due to their size, presence, and abundance in the area (Table S1). We used stringent conditions for peptide selection (see Materials and Methods section) and a sorting algorithm for protein assortment and taxon identification (Fig. 7).

**Fig 8:**
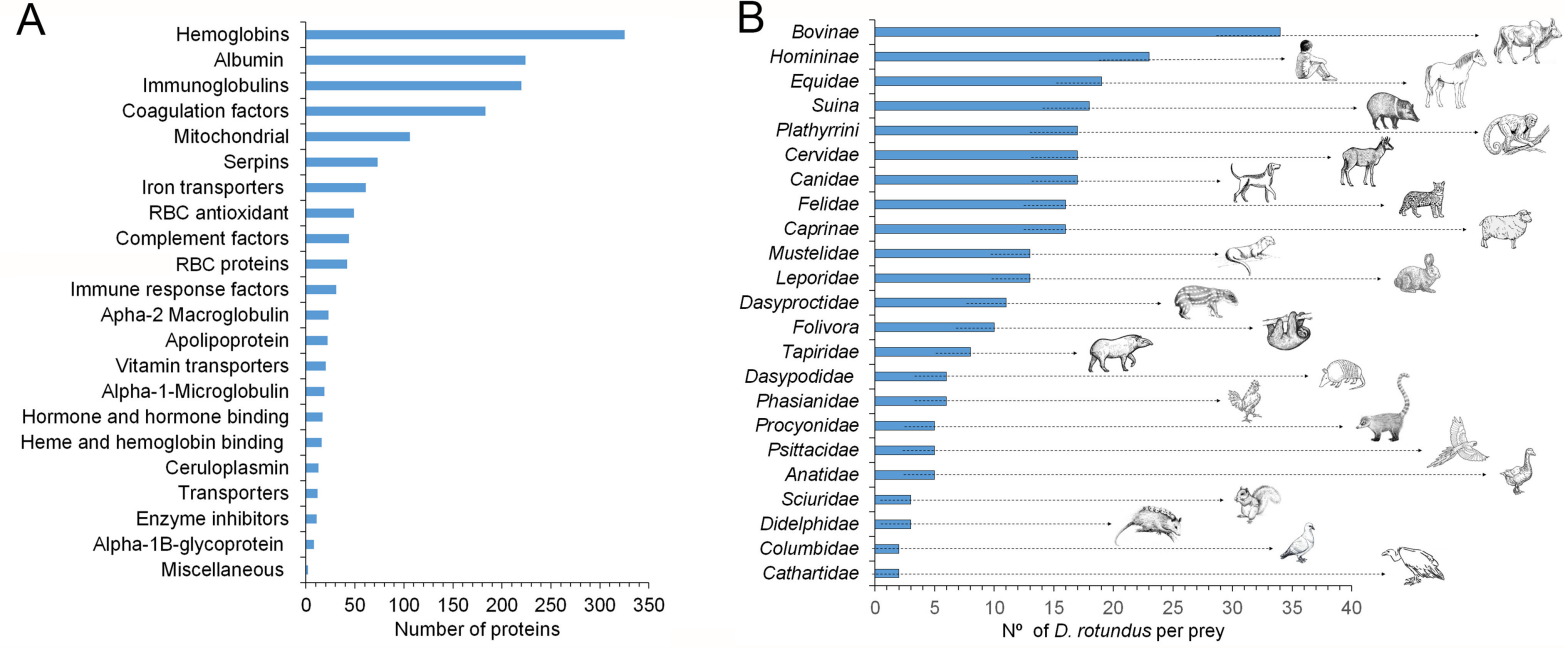
Proteins detected and taxa prey preferences of *D. rotundus* vampire bats. The number of plasma, red blood cell, and mitochondrial proteins was detected by proteomics in the gut content of the *D. rotundus* bat colony (**A**). The most likely foraged mammals were zebu and humans, which are highly abundant in the surroundings (40 - 45). Horses are part of the working force in the area. Collared peccary is in relatively high densities in the park, and suitable prey since the swine industry and homes with pigsties are absent in the area (Dataset S1). Spider and howler monkeys are abundant in the park and the natural reserves (42). Red brocket deer, relatively abundant, is the larger available prey in the forest. Dogs are popular in Costa Rica, and coyotes are abundant in Piedras Blancas National Park. Ocelots and cougars are more abundant than other felids in the park. Domestic cats are unlikely prey since they frequently hunt bats, including *D. rotundus*. Sheep are present in two farms in the surrounding, and goats are absent in the area. The park has abundant otters, forest rabbits, sloths, and agouti. Domestic rabbits are scarce in the area. Baird’s tapir is among the forest’s largest available prey. Armadillos, nosed coati, squirrels, and opossums are all mammals abundant in the park and have adapted to the suburban environments living close to houses. Domestic chickens and turkeys roaming around rural homes are frequent in the area. Other birds, such as ducks, geese, pigeons, and parrots, are abundant in the park (**B**).

Through the analysis of the available intestinal contents of 37 bats, a total of 54,508 peptides corresponding to 7,206 proteins were initially identified and finally sorted into 7,203 unique peptides corresponding to 1,019 plasma, 396 erythrocyte, and 106 mitochondrial proteins (Fig. 8A). Accordingly, 23 taxa prey groups were identified (Fig. 8B). Although presumptive for the moment, an educated guess of the genus/species prey preference can be assessed based on the abundance and accessibility of the various prey in the studied area (27 - 32). As expected, the most common prey were the larger and more abundant animals such as bovines, humans, and horses; but the choices were diverse. No vampire bat preference for either native or exotic animal species and vice versa was noted. Foraging on small mammals and birds seems unexpected, though there are reports of vampire bats chasing armadillos and feeding from squirrels, rats, chickens, among others (12, 38). Likewise, there have been cases of *D. rotundus* rabies virus variants transmitted by squirrels in Costa Rica, indicating that vampire bats also prey on these small mammals (39). In Costa Rica, nonindustrial poultry is usually kept in open chicken coops readily accessible to vampire bats attacks.

**Fig 9:**
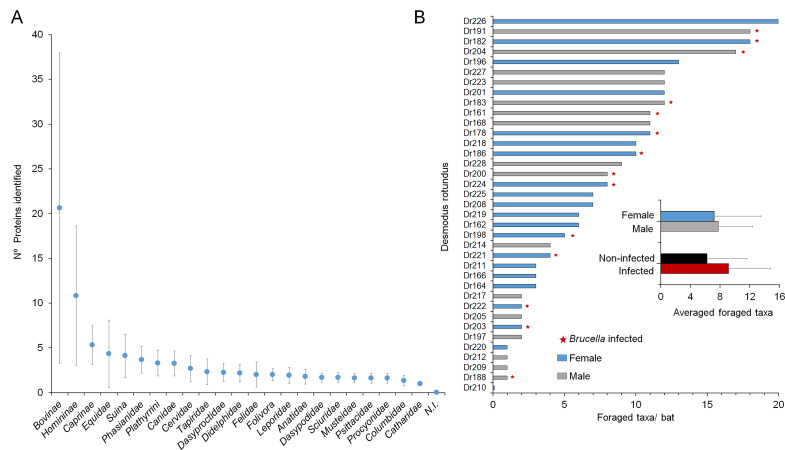
The number of proteins per identified taxa in the colony of vampire bats and the taxa prey number per *D. rotundus* studied. The average number of different proteins identified for each animal taxon was determined through proteomics of the intestinal content of 37 *D. rotundus* (**A**). Proteomic analysis identified the number of different animal blood taxa in each vampire bat’s gut (**B**). The red stars indicate the bats infected with *B. nosferati*. The inserted graph in (**B**) compares the average foraged taxa according to sex and *Brucella* infection status. No statistical differences between the sexes (*P* < 0.77) and infection status (*P* < 0.13) were found among the groups. Error bars are depicted.

The intestinal content of one vampire bat showed up to 20 different taxa prey groups. This variety is unlikely to mirror the foraging prey spectrum of single bats. Instead, it reflects the colony’s broad and diverse foraging range since vampire bats share their meals with their mates (19). Likewise, the fact that some bats are depicted with a reduced prey number, or none (Fig. 9A), does not mean they did not forage. Instead, the proteome in these animals was ambiguous, and unique peptides were not identified; therefore, they were not associated with a specific taxa group. There was no statistical difference (*P* < 0.13) between the number of prey foraged by individual bats with *Brucella* infection. Likewise, there was no statistical difference (*P* < 0.77) between *D. rotundus* sex and prey preference (Fig. 9A).

The number of proteins identified in each animal of the bat colony also indicates that cows, humans, and, to fewer extent, sheep, equines, and hog peccary (Suina) were among the preferred prey from which more blood was taken. The remaining animals were in the same range (Fig. 9B). However, the number of proteins in the bat’s intestinal content may be biased by the availability of data and influenced by the amount of blood taken and the time lapse between bloody meals.

## DISCUSSION

We found a high fraction (24%) of the colony of vampire bats infected with *B. nosferati*, being females infected in a higher proportion (65%) than males (35%). Due to the social behavior of vampire bats, it is plausible that once the bacterium reaches a colony, the infection is broadly distributed, paralleling what happens in ruminants (20). Like other pathogenic *Brucella* species, *B. nosferati* causes placentitis, induces fetal death, colonizes the mammary gland, is secreted in milk, causes orchiepididymitis, and is present in different tissues, all features of classical brucellosis (20) that can hamper the reproductive ability of bats. This pathology may have a reproductive impact on bat populations, considering that this pathogenic *Brucella* could potentially spread to other chiropterans since they often share roosts with other bat species (11).

It may be that the high livestock prey abundance favors *B. nosferati* parasitism in vampire bats. It has been shown that depending on the prey’s accessibility, the vampire bats’ innate and adaptive immune responses toward pathogens can shift (46). In regions of high-livestock accessibility, the microbicidal capacity of bats develops toward stronger innate immunity relative to adaptive response favoring more significant chronic stress. This shift in the immune response may affect the infection rate of bats to certain diseases, such as *Brucella* organisms whose immune control depends on a long-lasting adaptive immune response, as shown before (47, 48).

*B. nosferati* possesses all the required virulence arsenal of other zoonotic *Brucella* species and, therefore, can become a primary pathogen of animals and humans, as already demonstrated in nature. Since the bacterium can invade the salivary glands of bats, it is therefore feasible that *B. nosferati* could be transmitted between vampire bats and also to domestic and wildlife species, including humans. The *B. nosferati* BCCN84.3 strain causing dog orchiepididymitis (35, 36) supports this concept.

Considering that the *B. nosferati* was isolated from the bats’ gut contents of 47% of infected animals, we cannot dismiss the possibility that bats could have taken this bacterium from a third-party reservoir. We neither have attempted isolation in other potential hosts nor conducted a survey on antibodies against *Brucella* in wildlife. However, others have reported the presence of anti-*Brucella* antibodies in the blood of tropical wildlife mammals such as peccary, agouti, capybara, coati, raccoon, foxes, wild felids, otters, monkeys, and tyras (23, 49 - 52), all animal species identified in this work as prey of infected vampire bats. Moreover, the *B. nosferati* BCCN84.3-infected dog lived in an area inhabited by *D. rotundus* bats (34).

Of note is the recent description of an emerging *B. amazoniensis* infecting humans working in the deep Amazonian rainforest, which probably acquired the bacteria through contact with tropical wildlife, including bats (26). Although this bacterium is a different species, it branched closer to *B. nosferati* than other species. Likewise, a high seroprevalence in wild peccaries and capybaras was found in a tropical region of Venezuela with the concomitant isolation of organisms typed as *B. suis* using classical bacteriology (50, 53). However, conventional bacteriological typing cannot distinguish *B. suis* from *B. nosferati* (35). The closeness to areas with vampire bat attacks (40, 54) and the absence of pigs (*B. suis* reservoirs) in the vicinities where these sylvatic bacterial species were isolated (26, 50, 53) suggest the presence of zoonotic *Brucella* organisms in these tropical South American areas.

Our approach identified the feeding preferences of a *Brucella-*infected *D. rotundus* colony. Many studies through various decades have documented that *D. rotundus* feeds on several domestic and wildlife animals (6, 11, 12, 16, 18). In a single study, we identified the prey preference spectrum of a colony of *Brucella-*infected vampire bats in a given complex area, demonstrating the potential of our approach for control strategies in Latin America, where vampire bats thrive. This practice is particularly relevant when sudden outbreaks of infectious diseases such as rabies or brucellosis emerge in a given area, and the potential range of reservoirs requires fast identification for sanitary actions and epidemiological surveillance. Moreover, the same approach may be used to identify arthropod vector prey preferences.

It has been shown that many animals display defensive behavior against vampire bat attacks (41 - 43), escaping from their foraging range, partly explaining the bat colony’s wide prey range, including wild, dangerous predators and relatively small species of mammals and birds. Nevertheless, limitations in the availability of protein data for a given animal species cannot be circumvented. We aimed to diminish this bias by increasing specificity by selecting and curating major blood proteins since most taxa have pertinent information on these proteins. In addition, we broaden the chances by selecting higher taxa levels rather than attempting species or genus identification. However, by increasing specificity, the method’s sensitivity was diminished, and in some cases, we could not identify the taxa group unambiguously.

Vampire bats forage on humans in the surroundings of the Piedras Blancas National Park. Close to 35 bat bites in humans were reported in 2021 by the Costa Rican Ministry of Health (44). However, the number of bites is expected to be higher since these events are often unreported (45). Several investigators have also described a high incidence of vampire bats foraging on humans (up to 88% of the inhabitants) in tropical ecological conditions similar to those presented here (10, 17, 45, 55 - 59). Humans are abundant potential diurnal prey that have not developed natural defense mechanisms against vampire bat attacks when these chiropters hunt for blood at night. Bats frequently enter rural houses and shelters in tropical areas of Costa Rica (45, 60).

Considering the high prevalence of *Brucella* infections in *D. rotundus*, further studies are needed in prey victims to assess the zoonotic potential of *B. nosferati*. Unfortunately, in Latin America, including Costa Rica (22), bacterial isolation is seldom attempted in animals and humans, precluding the opportunity to identify the *Brucella* species (61).

It has been noticed that vampire bats reproduce better and in larger groups, precisely in those border areas where foraging is abundant, adjusting their immune response and favoring certain diseases over others (46). Following this, it is not surprising that *D. rotundus* is becoming a plague in certain areas where livestock and humans are abundant (62). Our recent experience suggests that bats may be reservoirs of novel and dangerous infectious diseases for humans and animals (63). Therefore, identifying a new species of pathogenic bacteria in *D. rotundus* vampire bats and establishing the range of potential hosts in a given environment are relevant for epidemiological studies and for preventing zoonotic infectious diseases, including brucellosis.

## MATERIALS AND METHODS

### Vampire bat collection and pathogen detection

Seventy-one *D. rotundus* were captured during the day using nets in a cave in the Pacific tropical rainforest in March 2020, Piedras Blancas National Park, Puntarenas, Costa Rica (8°43*'*40.1*''*N and 83°11*'*15.2*''*W) (Dataset S1). The bats were transported inside cotton bags to the Animal Health Service of Costa Rica, euthanized through intramuscular injection of xylazine and ketamine, and blood and organ samples were collected through systematic necropsies. Although vampire bats were collected as part of the National Surveillance Bovine Rabies Program of Costa Rica (see Ethics Statement), their blood and tissues were also screened for additional pathogen infections. Following this, the presence of coronavirus and rabies virus was explored through polymerase chain reactions as described (13, 64) and brucellosis by the rose bengal agglutination test (RBT) and cELISA for detecting antibodies against *Brucella* LPS (65, 66).

### Bacterial isolation and pathological studies

After necropsy, several tissues were tested for *Brucella* organisms (Table 2) following regular bacteriological procedures (65, 67, 68). Forty-two *Brucella* strains were isolated from the different organs of 17 *D. rotundus* vampire bats (Dataset S1). The placenta of a *D. rotundus* was subjected to histological studies following hematoxylin and eosin staining of the sections and immunolabeling of intracellular brucellae as described before (33, 67). Positive control tissue consisted of *B. abortus*-infected bovine placenta. No primary antibody was applied to the negative control. Several *Brucella* species were used as a reference.

### Bacterial phenotypic characterization

All *Brucella*-compatible colonies were analyzed as described (65). A biochemical profile was evaluated using the automated Vitek 2 System (bioMérieux) with the GN ID card to identify Gram-negative bacilli following the protocols described by the manufacturer. The vampire bat *Brucella* isolates and reference *Brucella* species, grown and harvested under the same conditions (Dataset S2), were examined by whole-cell MALDI-TOF proteomics and gas–liquid chromatography/ms (glc/ms) of the derived FAMEs as described before (35, 69, 70).

For MALDI-TOF, mass spectra were acquired using a VITEK MS Plus (bioMérieux), ranging from 2,000 to 20,000 Da at a laser frequency of 50 Hz. VITEK MS RUO (Research Use Only) database and SARAMIS software (bioMérieux) were used to capture spectra for each isolate. A consensus spectrum was built for reference strains and isolates. Principal component analysis was constructed using the Spearman correlation algorithm.

The glc/ms (Agilent Technologies 6850) was performed using a 25-m × 0.2-mm cross-linked phenyl-methyl silicone fused silica capillary column HP 19091B-102 (Agilent Technologies Inc., Santa Clara, CA, USA). Methyl ester determination was carried out according to the MIDI instruction manual of Technical Note #101 (MIS, MIDI Inc., Newark, DE, USA) as previously described (35, 69). The same culture and harvesting conditions for all bacteria were used during the experiment. Extraction of LPS was performed using the SDS-proteinase K method as described before (71). *Brucella* LPS was analyzed in 4%–10% gradient SDS-PAGE stained by the periodate-alkaline silver method or revealed by Western blotting with a monoclonal antibody against the A>M LPS epitope of the O-chain (72).

Dendrograms were constructed based on the absence or presence of peaks using an agglomerative hierarchical clustering algorithm, using XLSTAT 2022 (Version 1.2, 2022, Addinsoft, Inc., Brooklyn, NY, USA). The proximities were calculated using squared Euclidean distance and the aggregation using the unweighted pair group average method. One-way analysis of variance followed by Dunnett’s test or multivariate analysis of variance was used to determine statistical significance.

### DNA studies

DNA extraction, Bruce-ladder multiplex PCR, MLVA-16, and WGSA and Omp2b/Omp2a analysis were performed as described before (35, 69, 73 - 76). WGS was performed on Illumina platforms according to manufacturer protocol as described before (35, 69, 75). A representative sample (bbatCR03, Dataset S3 ) was also sequenced by Oxford Nanopore Technology (ONT). Quality control and adapter trimming were performed with bcl2fastq (https://support.illumina.com/sequencing/sequencing_software/bcl2fastq-conversion-software.html) and porechop (v0.2.4, https://github.com/rrwick/Porechop) for Illumina and ONT sequencing, respectively. Hybrid assembly with Illumina and ONT reads was performed with Unicycler (76). Assembly statistics were recorded with QUAST (77). All sequencing data were deposited at the National Center for Biotechnology Information (NCBI) under the accession codes listed in the dataset. The detailed information and metadata of the genomes used are presented in Dataset S3. SNPs were called using samtools (78), and 555,727 variable sites were extracted using SNP sites (79). The resulting alignment was used for maximum likelihood phylogenetic reconstruction with RAxML v8 (80). Genomes were aligned and mapped by bwa and SMALT v.0.5.8 using *B. suis* 1330 as a reference, with an average coverage of 93.50%. The phylogenetic tree was visualized with Figtree v1.4.3 (http://tree.bio.ed.ac.uk/software/figtree/) and edited with Dendroscope v3.5.8. The presence of recombination events was analyzed by Genealogies Unbiased By recomBinations In Nucleotide Sequences (81) and visualized by Phandango (82).

### Determination of the prey range of vampire bats

The prey range of *D. rotundus* vampire bats was estimated by species-oriented proteomic analysis of the intestinal content. The intestinal content, mainly constituted of tubular clots, was removed and placed in 1 mL sterile plastic tubes and frozen at −80°C until used. About 25 mg of intestinal content was suspended in 25 µL deionized double-distilled sterile water, gently stirred for 30 minutes, and then vortexed twice. The tubes were centrifuged at 12,000 rpm for 5 minutes, and the supernatants separated from the precipitate. Protein concentration was estimated using a Qubit Fluorometer (Life Technologies). Then, 130 µg of the 2-mercaptoethanol reduced proteins (5 minutes at 95°C) was separated by SDS-PAGE using 4%–20% precast gradient gels (Bio-Rad). Finally, the gels were stained with Coomassie blue R–250, as described elsewhere (83). The stained SDS-PAGE protein bands were excised from gels and subjected to reduction and subsequent enzyme digestion (83). The proteomic analysis was performed through nESI-MS/MS on a Q-Exactive Plus mass spectrometer (Thermo Fisher Scientific, Waltham, MA, USA) as described before (83). MS/MS spectra were processed to assign peptide matches to known protein families by similarity with sequences in the UniProt/SwissProt databases using Peaks X (Bioinformatics Solutions, Canada). Cysteine carbamidomethylation was set as a fixed modification, while deamidation of asparagine or glutamine and methionine oxidation were set as variable modifications, allowing up to two missed cleavages by trypsin.

Animal species identification was supported on the datasets constructed from Proteins-NCBI/UniProt/wiss-Prot (https://www.uniprot.org/) of the mammal and bird species found in the perimeter of the 8 km range where the *D. rotundus* bats were captured (Table S1). Due to the incomplete genomic and proteomic databases for most Costa Rican wildlife animals and birds, we complemented the data with the broader available information of the closest phylogenetic species for a given group (Table S1). The estimate was narrowed to 47 mammals and 14 bird species included in 25 taxa groups located within the foraging range of bats (27 - 32), making, in some cases, the identification at the species or genus level feasible (Table S1). Because of the close phylogenetic relationships of the species within target animal taxa, we expressed the identification at the subfamily, family, parvorder, or suborder level, according to the availability of the datasets. For example, in the case of the four opossum species (*Didelphidae*) present in Piedras Blancas National Park (27 - 32), the available databases were complemented with those of *Monodelphis domestica* and *Gracilinanus agilis* and other opossums from South America. Any target peptides for proteins of these South American animals were estimated to belong to one of the Costa Rican opossums. A similar approach was used for animal species from which data were insufficient. Likewise, the BLAST search for confirming human-derived peptides converged in some cases with apes and old-world monkey proteins such as chimpanzees, macaques, or papions, and therefore assigned to humans. Once the presumptive animal species was identified, it was assigned to a generic taxa-defined group (Table S1).

The data were curated as a final selection step by selecting only the most abundant plasma and red blood cell protein families (84) (Dataset S1). Due to their taxonomical value, major mitochondrial proteins were also included in the analysis. Extracellular, mucous-secreted, nuclear, and non-abundant red cell proteins were excluded from the study. Because of the close phylogenetic relationship among the various animal groups, we depicted the results at the nearest taxonomical rank identified above the genus level, such as family (e.g., Canidae and Felidae), subfamily (e.g., Bovinae and Caprinae), parvorder (e.g., Platyrrhini), or suborder (e.g., Suina and Folivora) (Table S1). For example, if one or more different presumptive proteins of *Felis catus* (domestic cat) and *Panthera onca* (jaguar) were identified, the match was considered positive and assigned to the family Felidae. This broader definition reduced the chances of taxonomic errors at the species or genus level. Whenever necessary, peptides were examined by a peptide-protein BLAST of the existing mammal and bird databases for unambiguous identification.

If a given protein species’ peptide matched a protein from a different generic taxon in the database, the peptide and the corresponding animal taxa were disregarded. Except for pigeons and squirrels, which are abundant in the park and surroundings, unlikely foraging vampire prey, such as chiropterans, animals of less than 600 g, aquatic birds and mammals, raptor birds, and nocturnal birds, were excluded from the analysis. *D. rotundus* proteome was included as a control for excluding peptides. File repetitions were removed from the datasets. The protein parameters for match acceptance were set to false discovery rate <0.1%, −10lgP protein score ≥50, and unique peptides for a given taxon were considered. Proteins of the respective animals were identified with the available GenBank protein database for the corresponding species (Dataset S1).

## Data Availability

All genomic data have been deposited in the National Center for Biotechnology Information (NCBI) under the accession numbers indicated in Dataset S3. All raw data are available upon request. These genetic resources were accessed in Costa Rica according to the Biodiversity Law #7788 and the Convention on Biological Diversity, under terms of respect to equal and fair distribution of benefits among those who provided such resources under CONAGEBIO Costa Rica permit # R-CM-UNA-005-2022-OT-CONAGEBIO.

## SUPPLEMENTAL MATERIAL

TABLE S1 msphere.00061-23-s0001.docx Animal species included in the proteomic databases to define the target taxa prey of *D. rotundus* in the Piedras Blancas National Park of Costa and the surrounding area.

Figure S1 msphere.00061-23-s0002.tif Phylogenetic analysis of the omp2a and omp2b nucleotide sequences of *B. nosferati* and other *Brucella* strains. *B. nosferati* grouped in a distinct cluster from other *Brucella* species.

Figure S2 msphere.00061-23-s0003.tif Recombination events in representative *Brucella* species. Each event is shown by a vertical block ordered along the genome.

Figure S3 msphere.00061-23-s0004.tif Presence and absence of anomalous regions or genomic islands in the *Brucella* genomes.

Dataset S1 msphere.00061-23-s0005.xlsx Metadata classification of *Desmodus rotundus* and *Brucella nosferati*, serology, organ selection, and taxa identification by proteomics of the vampire bat gut content.

Dataset S2 msphere.00061-23-s0006.xlsx Biochemical profiles, methyl-ester fatty acids, and MALDI-TOF analyses of *Brucella nosferati* isolates and reference strains.

Dataset S3 msphere.00061-23-s0007.xlsx Genomic data and metadata of *Brucella nosferati* strains and other Brucella for phylogenetic reconstruction and genomic analyses.

Supplemental Material Legends msphere.00061-23-s0008.docx Legends for the supplemental material.

ASM does not own the copyrights to Supplemental Material that may be linked to, or accessed through, an article. The authors have granted ASM a non-exclusive, world-wide license to publish the Supplemental Material files. Please contact the corresponding author directly for reuse.
