# Supplementary material for: Virulent Brucella nosferati infecting Desmodus rotundus has emerging potential due to the broad foraging range of its bat host for humans and wild and domestic animals
Source: mSphere. 2023 Jul 5;8(4):e00061-23. doi: 10.1128/msphere.00061-23 (PMC10449500; doi:10.1128/msphere.00061-23)
Supplement: TABLE S1 — Animal species included in the proteomic databases to define the target taxa prey of D. rotundus in the Piedras Blancas National Park of Costa and the surrounding area. [file msphere.00061-23-s0001.docx]

**Table S1.** Animal species included in the proteomic databases to define the target taxa prey of *D. rotundus* in the Piedras Blancas National Park and the surrounding area of Costa Rica^a^

| **Generic Taxon ^b^** | **Genus/species detected by proteomics ^c^** | **Likely target Costa Rican species ^d^** | **Common name of Costa Rican specie** |
| --- | --- | --- | --- |
| *Anatidae* | *Anser sp.* | *Anser anser* | Geese |
|  | *Anas sp.* | *Anas platyrhynchos** | Ducks |
|  | *Anseranas sp.* |  |  |
| *Bovinae* | *Bos sp.* | *Bos taurus* | European cattle |
|  | *Bubalus sp.* | *Bos indicus** | Zebu |
|  |  | *Bubalus bubalus* | Water buffalo |
| *Canidae* | *Canis sp.* | *Canis latrans** | Coyote |
|  | *Vulpes sp.* | *Canis lupus** | Domestic dog |
|  | *Urocyon sp.* | *Urocyon cinereoargenteus* | Gray fox |
| *Caprinae* | *Ovis sp.* | *Ovis aries** | Sheep |
|  | *Capra sp.* | *Capra hircus* | Goat |
| *Catharidae* | *Cathartes aura* | *Cathartes aura** | Turkey volture |
|  |  | *Coragyps atratus* | Black vulture |
| *Cervidae* | *Odocoileus sp.* | *Odocoileus virginianus* | White-tailed deer |
|  | *Cervus sp.* | *Mazama americana** | Red brocket |
|  |  |  |  |
|  |  |  |  |
| *Columbidae* | *Columba sp* | *Columba livia** | Common pigeon |
|  |  | *Patagioenas cayennensis* | Pale-vented pigeon |
| *Dasypodidae* | *Dasypus sp.* | *Dasypus novemincinctus** | Armadillo |
| *Dasyproctidae* | *Cavia sp.* | *Cuniculus paca** | Paca |
|  | *Dasyprocata sp.* | *Dasyprocta punctata** | Agouti |
| *Desmodontidae^e^* | *Desmodus rotundus* | *Desmodus rotundus* | Vampire bat |
| *Didelphidae* | *Didelphis sp.* | *Didelphis marsupialis ** | Black eared opossum |
|  | *Dasyurus sp.* | *Chironectes minumus* | Water oposum |
|  | *Monodelphis sp.* | *Philander opossum* | Graf four eye opossum |
|  | *Marmosa sp.* | *Marmosa alstoni* | Mouse opossum |
|  | *Philander sp* |  |  |
| *Felidae* | *Panthera sp.* | *Panthera onca* | Jaguar |
|  | *Puma sp.* | *Puma concolor** | Cougar |
|  | *Felis sp.* | *Leopardus pardalis** | Ocelot |
|  | *Herpailurus sp.* | *Leopardus wiedii,* | Magay cat |
|  |  | *Herpailurus yagouaroundi* | Jaguarundi |
|  |  | *Felis catus* | Domestic cat |
| *Folivora* | *Bradypus sp.* | *Bradypus variegatus** | Three-toed sloth |
|  | *Choloepus sp.* | *Choloepus hoffmanni* | Two-toed sloth |
| *Homininae* | *Homo sapiens* | *Homo sapiens** | Human |
| *Leporidae* | *Sylvilagus sp.* | *Sylvilagus brasiliensis** | Forest rabbit |
|  | *Oryctolagus cuniculus* | *Oryctolagus cuniculus* | Domestic rabbit |
| *Mustelidae* | *Lutra sp.* | *Lontra longicaudis** | Otter |
|  | *Lontra sp.* | *Galictis vittata* | Grison |
|  |  | *Eira barbara* | Tayra |
|  |  | *Conepatus semistriatus* | Skunk |
| *Phasianidae* | *Gallus gallus.* | *Gallus gallus** | Chicken |
|  | *Meleagris gallopavo* | *Meleagris gallopavo* | Turkey |
| *Platyrrhini* | *Ateles sp.* | *Ateles geoffroyi** | Spider monkey |
|  | *Cebus sp.* | *Alouatta palliata** | Howler monkey |
|  | *Saimiri sp.* | *Cebus capucínus* | Capucin monkey |
|  | *Callithrix sp* | *Saimiri oerstedii* | Squirrel monkey |
| *Procyonidae* | *Procyon sp.* | *Procyon lotor* | Racoon |
|  | *Nasua sp.* | *Nasua nasua** | Coati |
| *Psittacidae* | *Amazona sp.* | *Amazona farinosa** | Mearly parrot |
|  |  | *Ara ambiguous* | Green macaw |
|  |  | *Ara macao** | Scarlet macaw |
| *Sciuridae* | *Neosciurus sp.* | *Sciurus deppei** | Deppe's squirrel |
|  | *Sciurus sp* | *Sciurus granatensis* | Variegated squirrel |
| *Suina* | *Sus scrofa* | *Sus scrofa* | Domestic pig |
|  | *Tayassu sp.* | *Tayassu pecari* | White-lipped peccary |
|  |  | *Dicotyles tajacu** | Collared peccary |
| *Tapiradae* | *Tapirus terrestris* | *Tapirus barirdii** | *Baird's tapir* |
|  | *Diceros bicornis* |  |  |
|  | *Ceratotherium simum* |  |  |
| *Ramphastidae ^e^* | *Ramphastos sulfuratus* | *Ramphastos sulfuratus* | *Keel-billed toucan* |
|  |  | *Ramphastos ambiguous* | *Yellow-throated toucan* |
| *Vermilingua* ^e^ | *Cyclopes sp.* | *Cyclopes didactylus* | *Silky anteater* |
|  | *Myrmecophaga sp.* | *Myrmecophaga tridactyla* | *Giant anteater* |
|  | *Tamandua sp.* | *Tamandua mexicana* | *Collared anteater* |

*^a^ Cave location of* 8°43'40.1''N and 83°11'15.2''W, and the surrounding area in a radius of 8 km from the cave.

^b^ Generic taxons corresponded to either *Family*, *Subfamily*, *Pavorder,* or *Suborder*.

^c^ The closest phylogenetic species to Costa Rican species with available proteomic databases.

^d^ ( * ) Indicates the most likely target species of *D. rotundus* based on the relative abundance in the range of 8 Km (1-6)

^e^ No detected by proteomics.

Table S1 references.

1. Armin L, Walder C, Anton V, Timm E. 2008. Mammals of the Piedras Blancas National Park, Costa Rica:species composition, habitat associations and efficiency of research methods – a preliminary overview. Stapfia 88*:*409-422.

2. Avibase. 2022. Bird Checklists of the World, Piedras Blancas National Park. The World Bird Database https://avibase.bsc-eoc.org/checklist.jsp?region=CRpu06 Last visited March 4, 2023.

3. Beal MRW, Matzinger Saborio-R G, Noguera-Bristan J, Olson ER. 2020. Survey of medium-sized and large mammals of Piedras Blancas National Park, Costa Rica. Check List 16*:*939–950.

4. Carazo-Salazar JC, Hroutiounian T, Artavia A, Bone-Guzmán R. 2020. Ampliación del ámbito de distribución del coyote (*Canis latrans*) en la Península de Osa, Costa Rica. Rev Mex Mastozool, nueva época. 2:17-24.

5. Rodriguez-F J, Chinchilla FA. 1996. Lista de mamíferos de Costa Rica. Rev Biol Trop 44*:*877-890.

6. Wong G. 2014. Conservation status of large mammals on the Osa Peninsula, Costa Rica. PhD Thesis, University of Massachusetts Amherst, MA, AAI3615462 https://scholarworks.umass.edu/dissertations/AAI3615462.
