# Supplementary figures and images for: Virulent Brucella nosferati infecting Desmodus rotundus has emerging potential due to the broad foraging range of its bat host for humans and wild and domestic animals
Source: mSphere. 2023 Jul 5;8(4):e00061-23. doi: 10.1128/msphere.00061-23 (PMC10449500; doi:10.1128/msphere.00061-23)

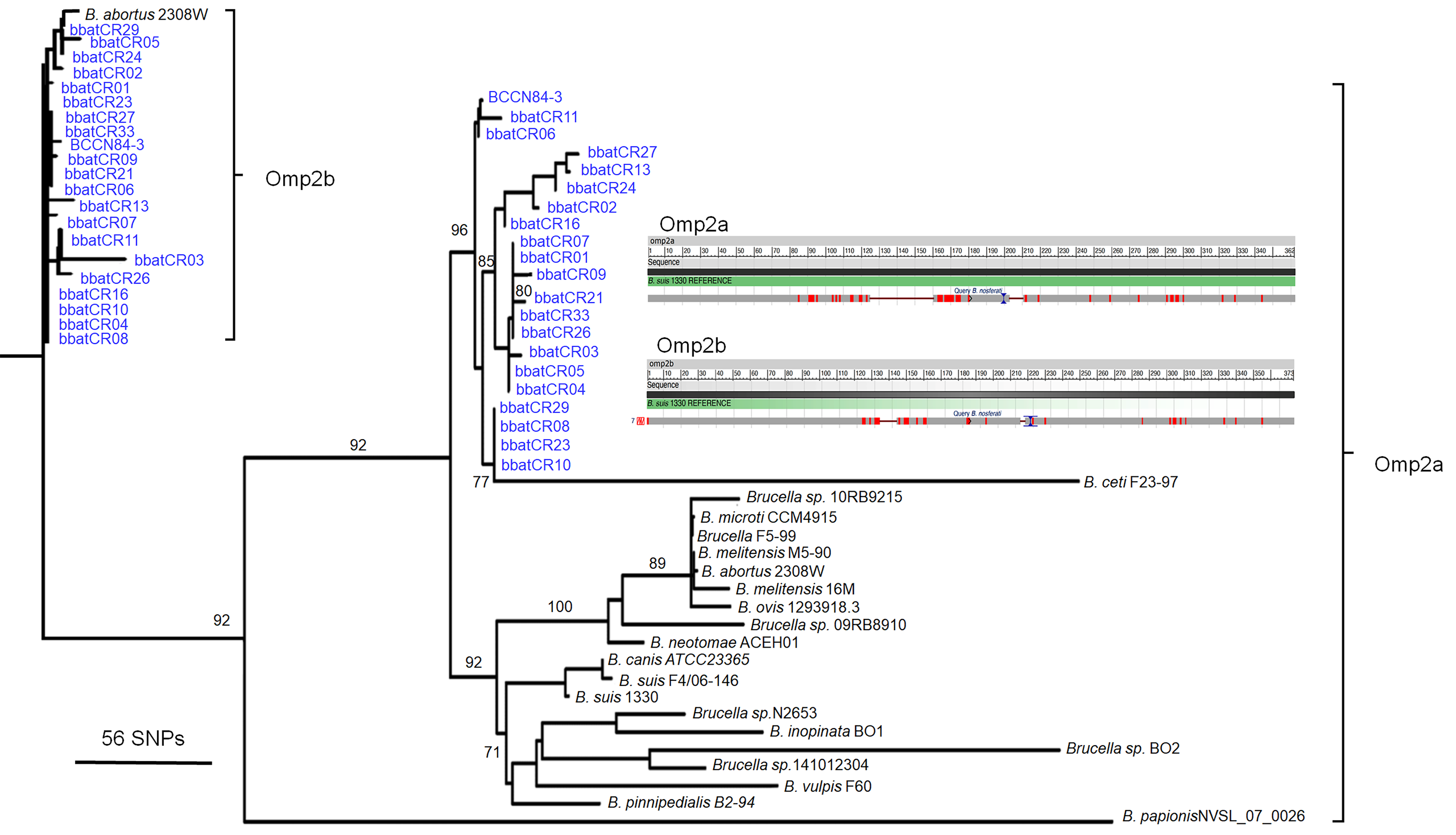

Supplement: Figure S1 — Phylogenetic analysis of the omp2a and omp2b nucleotide sequences of B. nosferati and other Brucella strains. B. nosferati grouped in a distinct cluster from other Brucella species. [file msphere.00061-23-s0002.tif]

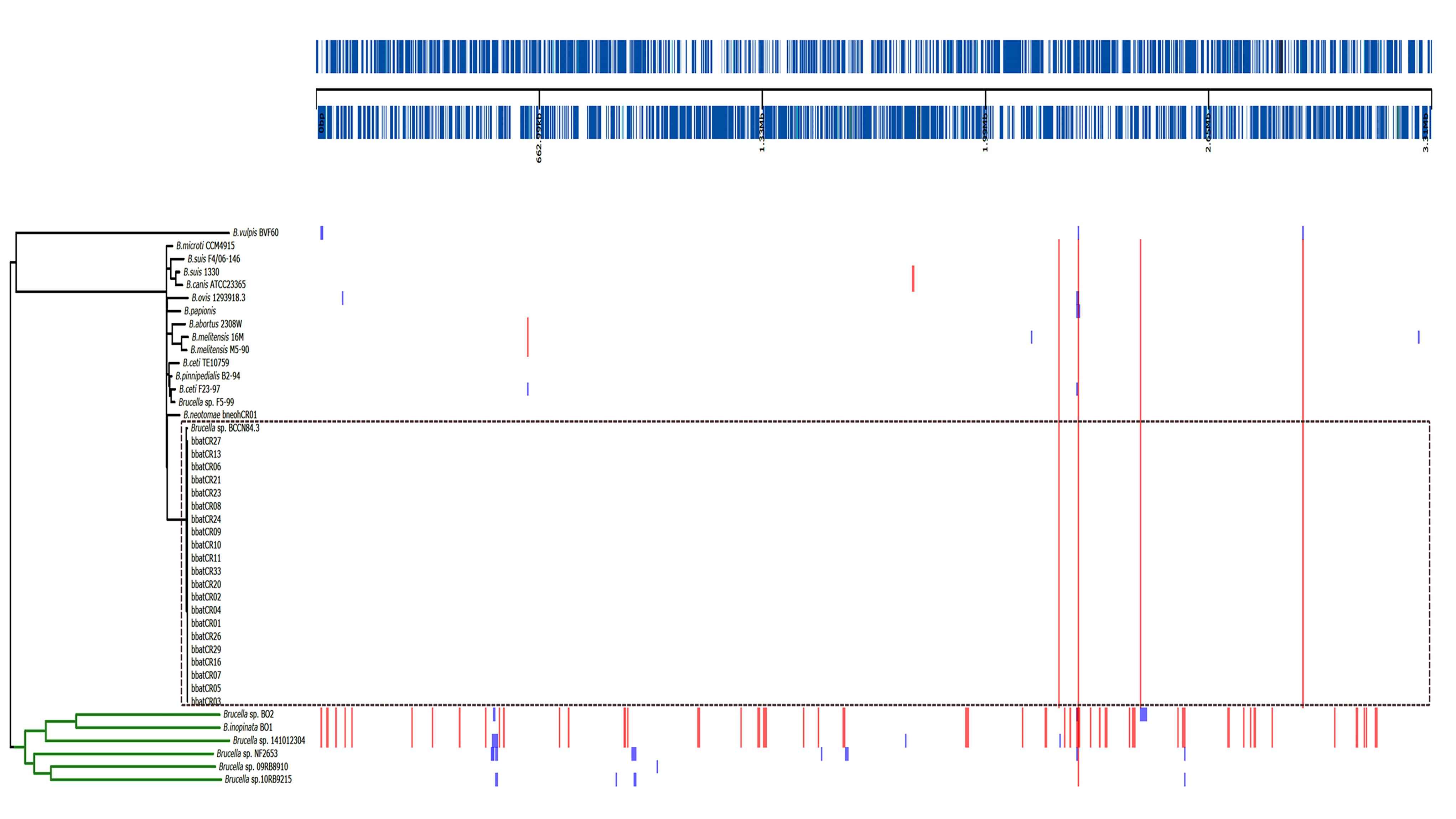

Supplement: Figure S2 — Recombination events in representative Brucella species. Each event is shown by a vertical block ordered along the genome. [file msphere.00061-23-s0003.tif]

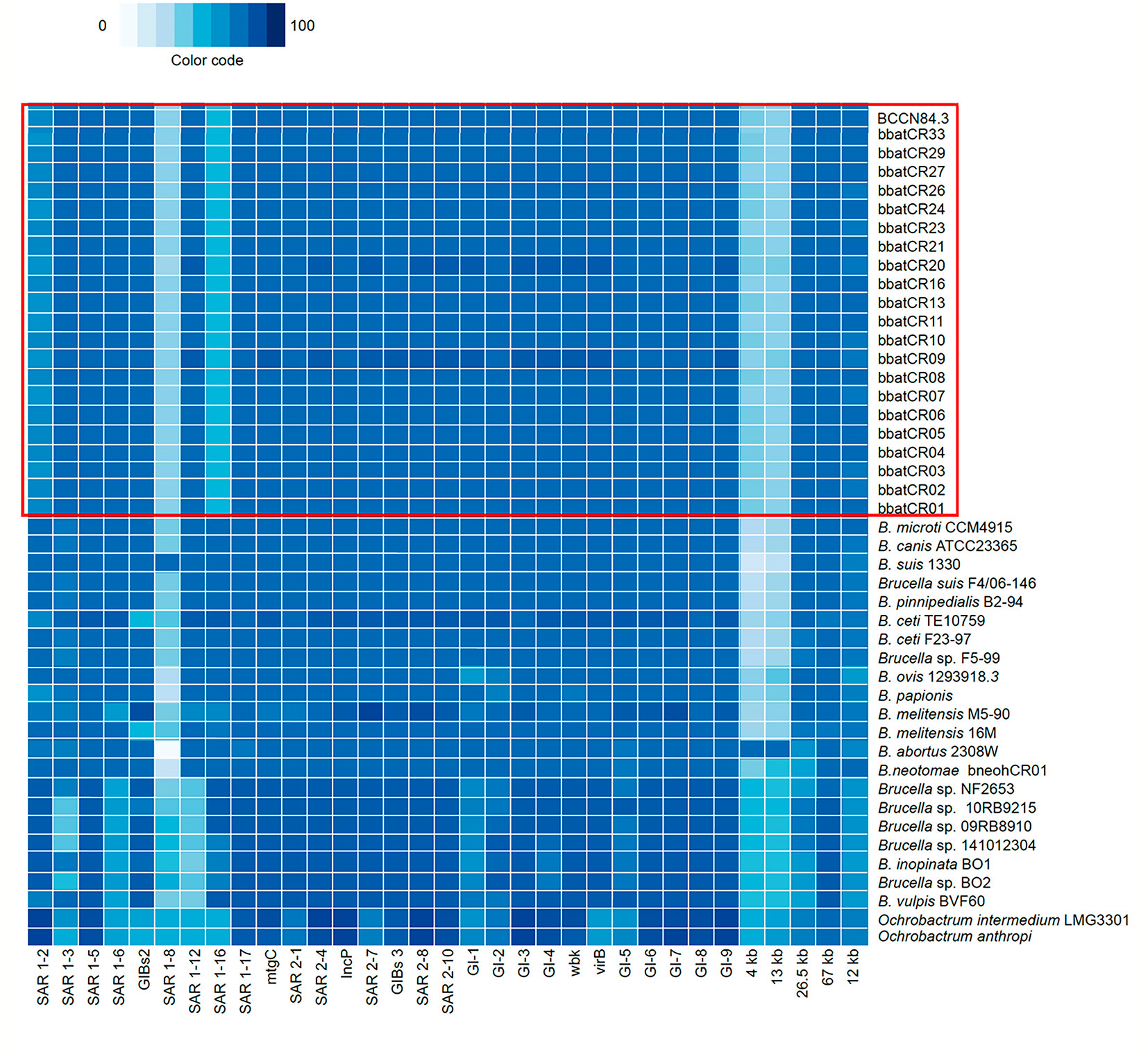

Supplement: Figure S3 — Presence and absence of anomalous regions or genomic islands in the Brucella genomes. [file msphere.00061-23-s0004.tif]
