# Supplementary material for: Virulent Brucella nosferati infecting Desmodus rotundus has emerging potential due to the broad foraging range of its bat host for humans and wild and domestic animals
Source: mSphere. 2023 Jul 5;8(4):e00061-23. doi: 10.1128/msphere.00061-23 (PMC10449500; doi:10.1128/msphere.00061-23)
Supplement: Supplemental Material Legends — Legends for the supplemental material. [file msphere.00061-23-s0008.docx]

SUPPLEMENTAL MATERIAL LEGENDS

Table S1. Animal species included in the proteomic databases to define the target taxa prey of *D. rotundus* in the Piedras Blancas National Park of Costa and the surrounding area.

FIG S1 Phylogenetic analysis of the omp2a and omp2b nucleotide sequences of *B. nosferati* and other *Brucella* strains. *B. nosferati* grouped in a distinct cluster from other *Brucella* species. The omp2a of *B. nosferati* isolates is somewhat closer to the omp2b cluster. Alignment of *B. nosferati* genome bbatCR03 omp2a and omp2b genes to the orthologous genes from reference *B. suis* 1330. SNPs are indicated as red bars and the blue vertical arrows represent deletions. For bacterial codes, see Supplemental data set 3.

FIG S2 Recombination events in representative *Brucella* species. Each event is shown by a vertical block ordered along the genome. The Upper black line represents the approximate coordinates in base pairs according to reference *B. suis* 1330; each blue line represents a coding sequence in the reference. Red blocks are recombination events shared by more than two genomes included in the analysis; blue blocks are unique. Classic *Brucella* species show few recombination regions; however, a higher number was detected in the non-classical clades. The *B. nosferati* clade is highlighted by a gray dotted line box.

FIG S3 Presence and absence of anomalous regions or genomic islands in the *Brucella* genomes. The upper left color scale represents the percentage of the island/region present in each genome; the darker blue indicates that the whole area is present. *B. nosferati* are highlighted by a red box.

Supplemental data set 1. Metadata classification of *Desmodus rotundus* and *Brucella nosferati*, serology, organ selection, and taxa identification by proteomics of the vampire bat gut content. Location and dates where and when vampire bats were captured in Piedras Blancas National Park cave, characteristics of B. nosferati isolates, serological results, and reference of genetic analysis performed (A). Farms located between 1.3 K and 5 Km from the Piedras Blancas National Park cave (B). Lists of the selected peptides and the corresponding proteins of the animal species identified by proteomics of the intestinal content of *Desmodus rotundus* vampire bats (C).

Supplemental data set 2. Biochemical profiles, methyl-ester fatty acids, and MALDI-TOF analyses of *Brucella nosferati* isolates and reference strains. *B. nosferati and* reference *Brucella* strains biochemical profiles (A). Fatty acid methyl esters identification of *B. nosferati* and reference *Brucella* strains. According to the cut of value, the data are presented as the presence (1) or absence (0) of the respective fatty acid for dendrogram construction (B). MALDI-TOF analyses of B. nosferati and reference *Brucella* strains. According to the cut-off, the data are presented as the presence (1) or absence (0) of protein mass values for dendrogram construction (C).

Supplemental data set 3. Genomic data and metadata of *Brucella nosferati* strains and other *Brucella* for phylogenetic reconstruction and genomic analyses. MLVA16 data for phylogenetic reconstruction of *B. nosferati* strains and other *Brucella* organisms (A). Metadata of WGS included in the phylogenetic reconstruction and for the *omps* *Brucella* trees (B). WGS SNPs Summary in comparison to *B. suis* 1330 as reference (C). Non-synonymous and synonymous substitutions in B. nosferati as compared to B. suis 1330 (D). The omp2a SNPs Summary in comparison to B. suis 1330 gene as reference (E). The omp2b SNPs summary in comparison to B. suis 1330 gene as reference (F).
